# Measuring patient experience: a systematic review to evaluate psychometric properties of patient reported experience measures (PREMs) for emergency care service provision

**DOI:** 10.1093/intqhc/mzx027

**Published:** 2017-03-02

**Authors:** Leanne Male, Adam Noble, Jessica Atkinson, Tony Marson

**Affiliations:** 1 University of Liverpool, Room 2.29, Clinical Sciences Centre, Aintree University Hospital, Fazakerley L9 7LJ, UK; 2 The Walton Centre NHS Foundation Trust, Lower Lane, Liverpool L9 7LJ, UK

**Keywords:** patient experience, emergency department, experience measure, PREM

## Abstract

**Purpose:**

Knowledge about patient experience within emergency departments (EDs) allows services to develop and improve in line with patient needs. There is no standardized instrument to measure patient experience. The aim of this study is to identify patient reported experience measures (PREMs) for EDs, examine the rigour by which they were developed and their psychometric properties when judged against standard criteria.

**Data sources:**

Medline, Scopus, CINAHL, PsycINFO, PubMed and Web of Science were searched from inception to May 2015.

**Study selection:**

Studies were identified using specific search terms and inclusion criteria. A total of eight articles, reporting on four PREMs, were included.

**Data extraction:**

Data on the development and performance of the four PREMs were extracted from the articles. The measures were critiqued according to quality criteria previously described by Pesudovs K, Burr JM, Harley C, *et al*. (The development, assessment, and selection of questionnaires. *Optom Vis Sci* 2007;**84**:663–74.).

**Results:**

There was significant variation in the quality of development and reporting of psychometric properties. For all four PREMs, initial development work included the ascertainment of patient experiences using qualitative interviews. However, instrument performance was poorly assessed. Validity and reliability were measured in some studies; however responsiveness, an important aspect on survey development, was not measured in any of the included studies.

**Conclusion:**

PREMS currently available for use in the ED have uncertain validity, reliability and responsiveness. Further validation work is required to assess their acceptability to patients and their usefulness in clinical practice.

## Background

Hospital Emergency Departments (EDs) assume a central role in the urgent and emergency care systems of countries around the world. Each and every patient attending ED should receive the highest quality of care. Currently, this is not always the case [1–4]. In the United Kingdom, for example, the 2014 Care Quality Commission report identified substantial variation in the care provided by EDs.

Patient experience is one of the fundamental determinants of healthcare quality [5]. Studies have demonstrated its positive associations with health outcomes [6–11]. Opening up dialogue between patients and providers by giving patients a ‘voice’ has proved key to improving quality of clincal experience [9]. Accordingly, there have been efforts made around the world to improve patient experience. In the UK, delivering high quality, patient-centred care has been at the forefront of health policy since 2008 [12, 13]. The UK government stated the importance of public involvement in prioritization of care needs and has recognized the significance of patient and public participation in the development of clinical services [14, 15]. It will only be possible to know if interventions and changes in practice are successful if processes and outcomes are measured.

To be able to identify where improvements in patient experience are required and to judge how successful efforts to change have been, a meaningful way of capturing what happens during a care episode is required. Patient reported experience measures (PREMs) attempt to meet this need. A PREM is defined as ‘a measure of a patient's perception of their personal experience of the healthcare they have received’. These questionnaire-based instruments ask patients to report on the extent to which certain predefined processes occurred during an episode of care [16]. For example, whether or not a patient was offered pain relief during an episode of care and the meaning of this encounter.

PREMs are now in widespread use, with both generic and condition-specific measures having been developed. The Picker Institute developed the National Inpatient Survey for use in the UK National Health Service. This PREM, which has been used since 2002, is given annually to an eligible sample of 1250 adult inpatients who have had an overnight stay in a trust during a particular timeframe. The results are primarily intended for use by trusts to help improve performance and service provision, but are also used by NHS England and the Department of Health to measure progress and outcomes.

Such ‘experience’-based measures differ from ‘satisfaction’-type measures, which have previously been used in an effort to index how care has been received. For example, while a PREM might include a question asking the patient whether or not they were given discharge information, a patient satisfaction measure would ask the patient how satisfied they were with the information they received. Not only are PREMS therefore able to provide more tangible information on how a service can be improved, they may be less to prone to the influence of patient expectation, which is known to be influenced by varying factors [17–21].

A number of PREMs have been developed for use within the ED. If the results from these PREMs are to be viewed with confidence, and used to make decisions about how to improve clinical services, it is important that they are valid and reliable. This means an accurate representation of patient experience within EDs (validity) and a consistent measure of this experience (reliability). If validity and reliability are not sound there is a risk of imprecise or biased results that may be misleading. Despite this, there has, to date, been no systematic attempt to identify and appraise those PREMs which are available for use in ED.

Beattie *et al*. [22] systematically identified and assessed the quality of instruments designed to measure patient experience of general hospital care [22]. They did not include measures for use in ED. This is important as there is evidence that what constitutes high quality care from a patient's perspective can vary between specialties, and by the condition, or conditions, that the person is being treated for [22–25]. Stuart *et al*. [26] conducted a study in Australia where patients were interviewed about what aspects of care mattered most to them in the ED. Patients identified the interpersonal (relational) aspects of care as most important, such as communication, respect, non-discriminatory treatment and involvement in decision-making [26]. This differs to what matters most to inpatients, where a survey in South Australia revealed issues around food and accommodation to be the most common source of negative comments and dissatisfaction [27].

This review aims to systematically identify currently reported PREMs that measure patient experience in EDs, and to assess the quality by which they were developed against standard criteria.

## Study objectives

The objectives of this review are as follows:

- To identify questionnaires currently available to measure patient experience in EDs.
- To identify studies which examine psychometric properties (validity and reliability) of PREMs for use in ED.
- Critique the quality of the methods and results of the measurement properties using defined criteria for each instrument.

Primarily, these objectives will lead to a clearer understanding of the validity and reliability of currently available instruments. This will support clinician and managerial decision-making when choosing a PREM to use in practice.

## Methods

### Eligibility criteria

Measure selection criteria were (i) description of the development and/or evaluation of a PREM for use with ED patients; (ii) instrument designed for self-completion by participant (or a close significant other, i.e. relative or friend); (iii) participants aged 16 years or older; (iv) study written in English.

Exclusion criteria were (i) studies focusing on Patient Reported Outcome Measures or patient satisfaction; (ii) review articles and editorials.

### Search strategy

Six bibliographic databases (MEDLINE, Scopus, CINAHL, PsycINFO, PubMed and Web of Science) were searched from inception up to December 2016. These searches included both free text words and Medical Subject Headings (MeSH) terms. The keywords used were ‘patient experience’ OR ‘patient reported experience’ OR ‘patient reported experience measure’; ‘emergency medical services’ (MeSH); ‘measure’ OR ‘tool’ OR ‘instrument’ OR ‘score’ OR ‘scale’ OR ‘survey’ OR ‘questionnaire’; and ‘psychometrics’ (MeSH) along with Boolean operators. Appendix 1 outlines the specific Medline search strategy used.

The Internet was used as another source of data; searches were conducted on Picker website, NHS surveys website and CQC, along with contacting experts in the field, namely at the Picker Institute. Finally, the reference lists of studies identified by the online bibliographic search were examined.

The search methodology and reported findings comply with the relevant sections of the Preferred Reporting Items for Systematic Reviews and Meta-Analyses (PRISMA) statement [28].

### Study selection

Articles were screened first by title and abstract to eliminate articles not meeting inclusion criteria. This was completed by two reviewers. Where a decision could not be made on the basis of the title and abstract, full text articles were retrieved.

### Data collection process

Using a standardized form, L.M. extracted the following information: name of instrument, aim, the target population, sample size, patient recruitment information, mode of administration, scoring scale, number of items/domains and the subscales used. This was also completed separately by J.A.

### Quality assessment tool

A number of frameworks exist to evaluate the quality of patient-reported health questionnaires and determine usability within the target population. This study utilized the Quality Assessment Criteria framework developed by Pesudovs *et al*. which has been used in the assessment of a diverse range of patient questionnaires [29–31].

The framework includes a robust set of quality criteria to assess instrument development and psychometric performance. The former includes defining the purpose of the instrument and its target population, the steps taken in defining the content of the instrument, and the steps involved in developing an appropriate rating scale and scoring system. The latter focuses on validity and reliability, as well as responsiveness and interpretation of the results. Some aspects of the Quality Assessment Criteria framework were relevant to development of questionnaires in which the patient reports on health status only rather than care experience. These were not considered when evaluating the PREMs.

Table 1 outlines the framework used to assess how the measure performs against each criterion. Within the study, each PREM was given either a positive (✓✓), acceptable (✓) or negative rating (X) against each criterion.

**Table 1:** Quality assessment tool

Property	Definition	Quality criteria
**Instrument development**		
Pre-study hypothesis and intended population	Specification of the hypothesis pre-study and if the intended population have been studied.	✓✓- Clear statement of aims and target population, as well as intended population being studied inadequate depth ✓- Only one of the above or generic sample studied X- Neither reported
Actual content area (face validity)	Extent to which the content meets the pre-study aims and population.	✓✓- Content appears relevant to the intended population ✓- Some relevant content areas missing X- Content area irrelevant to the intended population
Item identification	Items selected are relevant to the target population.	✓✓- Evidence of consultation with patients, stakeholders and experts (through focus groups/one-to-one interview) and review of literature ✓- Some evidence of consultation X- Patients not involved in item identification
Item selection	Determining of final items to include in the instrument.	✓✓- Rasch or factor analysis employed, missing items and floor/ceiling effects taken into consideration. Statistical justification for removal of items ✓- Some evidence of above analysis X- Nil reported
Unidimensionality	Demonstration that all items fit within an underlying construct.	✓✓- Rasch analysis or factor loading for each construct. Factor loadings >0.4 for all items ✓- Cronbach's alpha used to determine correlation with other items in instrument. Value >0.7 and <0.9 X- Nil reported
Response scale	Scale used to complete the measure.	✓✓- Response scale noted and adequate justification given ✓- Response scale with no justification for selection X- Nil reported
**Instrument performance**		
Convergent validity	Assessment of the degree of correlation with a new measure.	✓✓- Tested against appropriate measure, Pearson's correlation coefficient between 0.3 and 0.9 ✓- Inappropriate measure, but coefficient between 0.3 and 0.9 X- nil reported or tested and correlates <0.3 or >0.9
Discriminant validity	Degree to which an instrument diverges from another instrument that it should not be similar to.	✓✓- Tested against appropriate measure, Pearson's correlation coefficient <0.3 ✓- Inappropriate measure, but coefficient <0.3 X- Nil reported or tested and correlates >0.3
Predictive validity	Ability for a measure to predict a future event.	✓✓- Tested against appropriate measure and value >0.3 ✓- Inappropriate measure but coefficient >0.3 X- Nil reported or correlates <0.3
Test-retest reliability	Statistical technique used to estimate components of measurement error by testing comparability between two applications of the same test at different time points.	✓✓- Pearson's *r* value or ICC >0.8 ✓- Measured but Pearson's *r* value or ICC <0.8 X- Nil reported
Responsiveness	Extent to which an instrument can detect clinically important differences over time.	✓✓- Discussion of responsiveness and change over time. Score changes > MID over time ✓- Some discussion but no measure of MID X- Nil reported

Each PREM was independently rated by two raters (L.M., J.A.) against the discussed criteria. Raters were graduates in health sciences who had experience in PREM development and use. They underwent training, which included coding practice, using sample articles. Once the PREMs had been rated, any disagreements were resolved through discussion.

## Results

### Study selection

**Figure 1:**
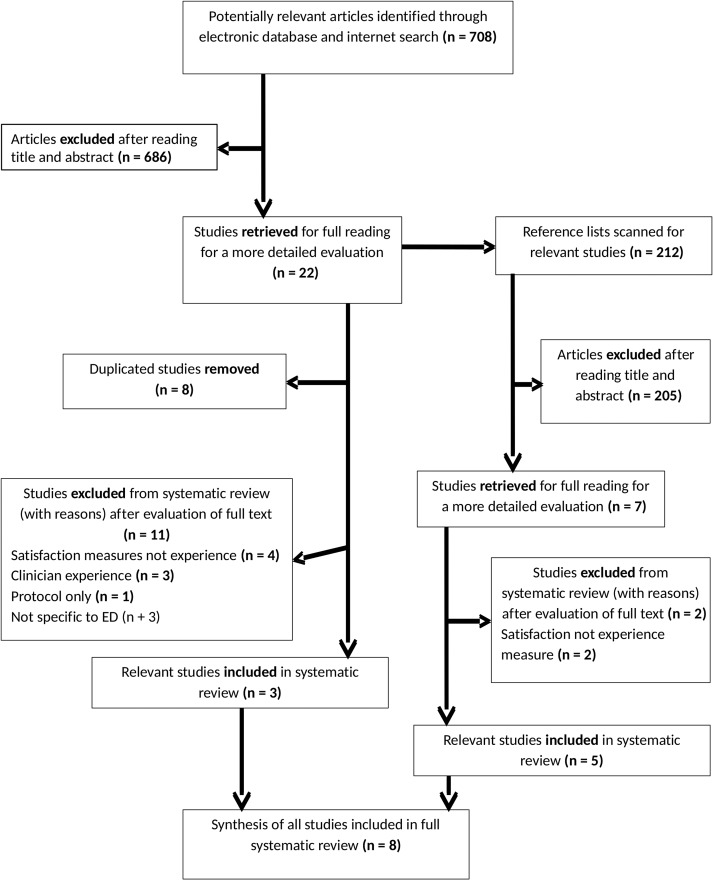
Selection process flow diagram.

Study selection results are documented with the PRISMA flow diagram in Fig. 1. A total of 920 articles were identified, of which 891 were excluded. Full text articles were reviewed for the remaining 29 articles, after which a further 21 articles were excluded for the following reasons: duplication of same publication (*n* = 8), patient satisfaction measure rather than experience (*n* = 6), protocol only (*n* = 1), clinician experience measure (*n* = 3) and PREM not specific to ED (*n* = 3). A total of eight papers met the inclusion criteria representing four different PREMs.

### Characteristics of included studies

Study characteristics are summarized in Table 2. All eight studies were conducted after 2008 within Europe. Three studies described the development of a PREM using qualitative data to elicit concepts. The other five studies evaluated psychometric development of the PREMs. Four were original studies and one further evaluated and developed the psychometric testing of an original instrument [32]. Within these four original studies there was variety in the recruitment process. Two were multicentre studies in hospital trusts [33, 34], one targeted a single specific hospital trust [35] and one recruited through general practice [36]. All five studies assessing the psychometric development of PREMs had over 300 participants with a mean age range of 51–56. Not all measures reported specific age ranges and one did not discuss participant demographics [37]. Two of the studies recruited using purposive sampling [33, 34], one through a systematic random sample [35] and one used a geographically stratified sample combined with random digit dialling for telephone surveys [37].

**Table 2:** Data extraction results

Reference	PREM developed	Research aim(s)	Qualitative method	Participants, sample selection and socioeconomic (SE) factors	Setting	Main themes
Picker Institute Europe [38]	Bos *et al*. [35] Accident and Emergency Department Questionnaire (AEDQ)^a^	Define the sampling framework and methodology that would be usable in all NHS acute trusts using emergency care. To identify issues salient to patients attending ED. Consult with project sponsors regarding scope of survey. Test the face validity of the questionnaire using cognitive interviews.	Focus groups (*n* = 4)	35 participants—male (*n* = 17), female (*n* = 18). Recruitment by specialist research recruitment agency—purposive recruitment based on age, sex and area of residence. Participants must have attended ED within the last 6 months. A selection was made with regard to socioeconomic status (based on present or most recent occupation).	ED attendance in one of two locations in UK:- Large sized city (3 different EDs) (*n* = 20) Medium-sized coastal town (2 different EDs) (*n* = 15)	**Waiting** Length of time to be seen. Being told how long they would be waiting. Waiting time at different stages (i.e. waiting for tests, waiting for results) **Staff-interpersonal aspects of care** Having confidence and trust in staff. Being treated with dignity and respect. Being able to understand explanations given by nurses and doctors. Doctors and nurses listening carefully to patients. **Tests and Treatment** Assessing pain and providing pain relief (particularly while still waiting to see the doctor). Condition/injury dealt with to patient's satisfaction. Not receiving conflicting advice from staff. Not having to return to ED following day due to visit being ‘out of hours’ for tests/treatment. **Environment** Level of privacy at reception when ‘booking in’. Cleanliness of ED. Not feeling disturbed or threatened by other patients. Overall comfort of waiting areas. **Discharge or admission to a bed** Being given information about their condition and/or treatment. Being admitted to a bed on a ward quickly and/or not having to wait too long to be transferred to another hospital. **Other issues** Reason for attending ED as opposed to other services (e.g. minor injuries unit, NHS Direct , GP, etc.). Car parking. **Being acknowledged**
Frank *et al*. [36]	Frank *et al*. [33] Patient Participation in the Emergency Department (PPED)	Describe patients’ different conceptions of patient participation in their care in an ED.	Interviews (*n *= 9)	9 participants—women (*n* = 4), men (*n* = 5). Purposive strategic sampling based on sex, age and patients from different sections of the ED (i.e. medical, surgical, infectious diseases, orthopaedics, and ear, nose and throat).	One ED in a metropolitan district in Sweden	**Having a clear space** **Struggling to become involved**
O'Cathain *et al*. [39]	O'Cathain *et al*. [37] Urgent Care System Questionnaire (UCSQ)	To explore patients views and experiences of the emergency and urgent care system to inform the development of a questionnaire for routine assessment of the systems performance from the patient's perspective.	Focus groups (*n* = 8) and interviews (*n* = 13)	60 participants—8 focus groups with 47 participants and 13 individual interviews. Purposive sampling of focus groups—covering a range of demographic and geographic groups. This included parents of young children, people with no children, a group socially deprived, an affluent group, another of black and ethnic minority people, a group living in a rural area and one living in an urban area. Approached face-to-face in the street and invited if they had an urgent health problem in the past 4 weeks and attempted to contact any service within the emergency and urgent care system. For individual interviews, recruitment was done through a GP practice in one primary care trust. A purposive sample was selected by a GP or other member of practice staff based on inclusion criteria provided	Patients treated across various ED and other emergency care services in UK. Focus groups completed in localities of Yorkshire to ensure participants could attend. Interviews conducted in participants own homes.	**Seeing the System** **Accessing the System** Choice or confusion? Making choices. Ease of access. **Communication and Coordination** Coordination between services. Informational continuity—the importance of patient records. Communication between professionals and patients. The effect of waiting—a vacuum of information. **Progress through the system** Need for proactive behaviour. Seeking healthcare in the context of social responsibilities.

Reference	Research aim(s)	Mode of administration	Participants, sample selection and socioeconomic (SE) factors	No. of items/domains in measure	Domains measured
Bos *et al*. [35] Accident and Emergency Department Questionnaire (AEDQ)^a^	To determine which method of obtaining summary scores for the A&E department questionnaire optimally combined good interpretability with robust psychometric characteristics	Self-completion postal questionnaire	151 hospital trusts in England. For each eligible trust, a systematic sample of patients out of a 1-month sample of A&E attendees was selected (*n* = 850). Patients not eligible is they were <16 years old, had attended minor injuries unit or walk-in centre, had been admitted directly to Medical or Surgical Admissions Units or had planned attendance at outpatient clinic run through the emergency department. Patients not eligible is they were <16 years old, had attended minor injuries unit or walk-in centre, had been admitted directly to Medical or Surgical Admissions Units or had planned attendance at outpatient clinic run through the emergency department. Age range: 54 (mean) Gender: 45% male, 55% female Ethnicity: Not discussed	51 items; 11 domains	Arrival at Emergency Department (5 items) Waiting (4 items) Doctors and Nurses (7 items) Your care and treatment (6 items) Tests (4 items) Pain (4 items) Hospital environment and facilities (3 items) Leaving the emergency department (8 items) Overall (2 items) About you (8 items) Any other comments
Frank *et al*. [33] Patient Participation in the Emergency Department (PPED)	To develop and test the psychometric properties of a patient participation questionnaire in emergency departments	Self-completion postal questionnaire	356 participants recruited after 4 reminders (46% uptake). ED patients over 3 days at three hospitals in central Sweden (28–30 Nov. 2008). Eligibility not specific; over 18 years. Exclusion criteria were those accompanied by an interpreter and those registered as deceased during the ED visit. Age range: 19–94 (56 mean) Gender: 51% male, 49% female Ethnicity: Not discussed	17 items; 4 domains	Fight for participation (5 items) Requirement for participation (5 items) Mutual participation (4 items) Participating in getting basic needs satisfied (3 items)
O'Cathain *et al*. [37] Urgent Care System Questionnaire (UCSQ)	To psychometrically test the Urgent Care System Questionnaire (UCSQ) for the routine measurement of the patient perspective of the emergency and urgent care system	Self-completion postal questionnaire and telephone survey	Response rate to postal survey (*n* = 457) (51% uptake). In telephone survey—11 604 calls made to obtain quota sample of 1014. The 2 surveys identified *n* = 250 participants who had used system within previous 3 months. Postal survey of 900 of the general population and telephone survey of 1000 members. Selected randomly by geographical stratified sample and random digit dialling. Sent directly to patients over 16 years and to parents/guardians of those under 16 years of age. No specific eligibility criteria apart from patients must have used emergency or urgent care system within the previous 3 months. Age range: Not discussed Gender: Not discussed Ethnicity: Not discussed Conducted in UK	21 items; 3 domains	Progress (13 items) Entry (3 items) Patient Convenience (5 items)
Bos *et al*. [34] Consumer Quality index of the accident and emergency department (CQI-A&E)^a^	Development of a patient reported experience measure for accident and emergency departments—Consumer Quality index of the accident and emergency department (CQI-A&E)^a^	Self-completion postal questionnaire	Discussion of content development within this study as there was no previous qualitative study conducted. Content Development: Focus groups with patients (*n* = 17) treated in the A&E department at the University Medical Centre Utrecht, aged 18 and over, with known postal address and phone number were sent invitation by post to participate. (*n* = 10) also took part in cognitive interviews—no eligibility/selection criteria noted. Psychometric validation: Large urban hospital in central location in Netherlands. All patients who attended A&E during one week in January 2010 were included (*n* = 653). Patients who attended with known postal address and no reported death were eligible. Participants *n* = 304 (47% uptake) Age range: 51 (mean) Gender: 159 male, 145 female Ethnicity: Not recorded	84 items; 9 domains	General Before arriving in the A&E Reception desk A&E Health professionals in the A&E Pain Examination and treatment Leaving the A&E General A&E About you
Bos *et al*. [32] Consumer Quality index of the accident and emergency department (CQI-A&E)^a^	To test internal consistency, the validity and discriminative capacity of CQI-A&E^a^ in a multicentre study design, to confirm and validate preliminary results from Bos *et al*. [34].	Self-completion postal questionnaire	Total of 4883 of participants responded (40% uptake). Announcement made in online medical national newsletter in Netherlands. 21/100 EDs in Netherlands chose to participate. In the sample, 600–800 patients per ED were selected randomly out all ED attendances in the previous 3 weeks. Patients with a known postal address and no reported death were eligible. Age range: 52.8 (mean) Gender: 49% male, 51% female Ethnicity: Not discussed	78 items; 9 domains	General (3 items) Before arriving in the A&E (11 items) Reception desk A&E (4 items) Health professionals in the A&E (8 items) Pain (3 items) Examination and treatment (16 items) Leaving the A&E (11 items) General A&E (11 items) About you (11 items)

^a^Accident and Emergency (A&E) used interchangeably with Emergency Department (ED).

All of the studies utilized postal self-completion questionnaires [33–35,37], with the Urgent Care System Questionnaire (UCSQ) also incorporating telephone surveys [37]. The length of the PREMs described within the studies varied from 17–84 items across 3–11 domains. Domain contents and names varied, as detailed in Appendix 3, but did cover characteristics identified by the Department of Health [5]. Half focused on the sequential stages of the hospital episode [34, 35], whereas others focused on specific areas of care, such as patient participation [33] and convenience [37]. All instruments were administered following discharge from hospital but the time from discharge to completion varied between measures.

### Instrument development and performance

A summary of the instrument development is presented in Table 3. All of the measures reported aspects of psychometric testing with evidence that validity was tested more frequent than reliability. Content validity was reported on most often.

**Table 3:** Quality assessment of PREMs

Measure	Pre-study hypothesis/ intended population	Actual content area (face validity)	Item identification	Item selection	Unidimensionality	Choice of Response Scale
**Instrument development**						
CQI-A&E [34] Consumer Quality Index Accident and Emergency	✓✓	✓✓	✓✓ Questionnaire focus groups were conducted with 17 participants and a further 10 participants were involved in the cognitive interviewing process [34].	✓✓ Clear explanation of how missing items were handled. Questionnaire was excluded if it was returned with over 50% missing items.	✓✓ Cronbach's alpha >0.7 in all domains.	✓ Likert scale used but 2,3,and 4 point scales used. No justification is given as to why such a variety of scales were used within the same measure.
AEDQ [35] Accident and Emergency (A&E) Department Questionnaire	✓✓	✓✓	✓✓ The Department of Health and Healthcare Commission were consulted. Focus group interviews with patients were completed with 35 participants over 4 focus groups. The draft questionnaire was tested using cognitive interview techniques [38].	✓✓	✓ A&E department questionnaire had 13 domains, 6 of which had an *α* < 0.70 demonstrating reduced unidimensionality.	X
PPED [33] Patient Participation in Emergency Departments	✓✓	✓✓	✓✓ Questionnaire was created following phenomenological analysis of 9 depth interviews with patients who had previously been treated in an ED. Concepts generated through data analysis were used to develop questions. [36]	✓✓	✓✓ Cronbach's alpha of 0.75 during first test and 0.72 from second test but two of the four domains had an *α* < 0.70	✓✓
UCSQ [37] Urgent Care System Questionnaire	✓✓	✓ Assessed by cognitive testing of the measure in earlier qualitative research and by checking for consistency of answers with each questionnaire.	✓ Content validity was derived from basing the questionnaire development on previous qualitative research (consulting with patients) Focus groups were completed with 47 people and 13 individual interviews purposively selected from GP practices in one geographic area [38]. A literature review was also conducted as part of this process.	✓ Missing values for postal and telephone surveys ranged from 0 to 4%. This was much higher for satisfaction questions at 12–18%. Some respondents put ‘N/A’ against answers, demonstrating that a ‘does not apply’ option was necessary, as some questions were only relevant to some participants. Interpretation of ceiling effects identified a positive skew for telephone survey over postal survey. This may be due to social desirability bias.	✓✓ Cronbach's alpha > 0.7 in all domains.	✓✓

Measure	Convergent validity	Discriminant validity	Predictive validity	Test-retest reliability	Responsiveness
**Instrument performance**					
CQI-A&E [34] Consumer Quality Index Accident and Emergency	X	✓ Discussion is had around discriminative capacity between different EDs in different hospitals. All 5 domains regarding quality of care and the ‘global quality rating’ had capacity to discriminate among EDs	X	X	X
AEDQ [37] Accident and Emergency (A&E) Department Questionnaire	✓ Convergent validity measured with Pearson's coefficient although not measured against a separate measure. The study measured the overlap in concepts within the same measure.	X	X	X	X
PPED [33] Patient Participation in Emergency Departments	X	X	X	✓ Intraclass coefficient measured for test-retest reliability. This varied between 0.59 and 0.93, therefore was not always within statistical limits. It is noted within the paper that there was a low response rate so this therefore may have had effect on the results.	X
UCSQ [35] Urgent Care System Questionnaire	X	X	X	X	X

✓✓- positive rating, ✓- acceptable rating, X- negative rating.

The key patient-reported concepts that were incorporated into the quantitative measures through item selection included waiting time, interpersonal aspects of care, tests and treatment, and the environment. Qualitative concept elicitation work revealed similar concepts that were most important to patients [36, 39]. CQI-A&E also conducted an importance study to establish relative importance of items within the questionnaire to patients visiting the ED [34]. All measures addressed very similar themes under varying headings.

Item selection was generally well reported with adequate discussion of floor/ceiling effects. Likert scales were used in all bar one study [35], where choice of response scale was not discussed.

Quality appraisal of instrument performance demonstrated a limited level of information on construct validity, reliability and responsiveness throughout all four measures.

All instruments demonstrated the use of unidimensionality to determine homogeneity among items. Of the four measures identified, not one study assessed the responsiveness by measuring minimal clinically important difference.

## Discussion

### Methodological quality of the instruments

To our knowledge, this is the first systematic review to identify PREMs for use in the ED and evaluate their psychometric properties. Four PREMs were identified and subjected to an appraisal of their quality. While the developers of each measure reported them to be valid and reliable, the quality appraisals completed within this review do not fully support this position. Further primary studies examining their psychometric performance would be beneficial before the results obtained can be confidently used to inform practice.

Content validity and theoretical development have been well reported across all four PREMs. Item generation through patient participation is important to determine what quality of care means to local populations. It is imperative, however, that it is recognized and this may vary across populations. For example, work carried out to find out what matters to patients in the concept elicitation phase of UCSQ [39] was completed in the UK. If this instrument was to be used in another country, then studies of cross-country validity would have to be completed before using the questionnaire.

Validity and reliability are not an inherent property of an instrument and should be addressed in an iterative manner throughout development. Often, validity and reliability changes over time, as refinements are made. Instrument validity and reliability should be reassessed throughout development to ensure the overall performance is not altered. For example, there are previous versions of the AEDQ dating back to 2003. However, there are validation papers for survey development up until 2008 [38] where focus groups are used to discuss what is important to patients. It is important to keep up to date with changes, as relying on past data can render an instrument poor in terms of validity.

Furthermore, issues around validity of the instrument can change dependent on the data collection process. For example, the UCSQ used both postal and telephone survey to collect data. However, there was no discussion of validation of the PREM for use in both methods.

Disappointingly, for none of the PREMs studied did we find evidence on responsiveness. Responsiveness refers to the ability of an instrument to detect change over time. This is a highly relevant factor if a PREM is to be used to assess how successful an intervention has been to enact change within a service [13]. This review highlights the current gap in studies assessing the responsiveness of PREMs, which should be addressed.

Some instruments appear to have limited positive psychometric properties and caution should be taken when using such measures. This is not to say that these instruments do not have their uses but careful consideration should be taken when selecting an instrument.

Using Pesudovs criteria for quality assessment [29] offered a rigorous and standardized critique of validity and reliability. At times it appeared difficult to fit particular psychometric results into the quality criteria used. For example, CQI-A&E used an important study as part of content validity which did not fall agreeably into any particular quality criteria category. We used consensus discussion to reach agreement on anomalies within the data. Pesudovs criteria prove to be a good starting point for assessing psychometric properties of PREM development.

### Strengths and limitations of this review

Application of the search strategy identified four PREMs that fitted the inclusion criteria. This low number was expected considering the current advances in the importance of patient experience measures within healthcare and the specificity of the population of an ED. It may be that not all PREMs were identified in the search, but scoping searches and reference list searches attempted to address this issue. Poor reporting and inadequate abstracts may have led to PREMs being erroneously left out in some cases; however, a representative sample has been included.

Data extraction of papers not included in the study was completed by both the main author (L.M.) and supervisor (A.N.) to cross-check data extraction and quality appraisal process. Papers containing PREMs not included in the study were selected to reduce bias in findings. This process allowed assessment of the rate of agreement prior to data extraction of the studies included in the review. Data extraction of studies included within the review was conducted by L.M. and J.A..

### Interpretation of findings in relation to previously published work

There is little evidence of similar reviews evaluating the psychometric properties of PREMs for emergency care. Findings regarding the limited information about the reliability and validity of the measures within the general population are supported by outcomes of a recent evidence review conducted by The Health Foundation [40]. This research recognized that hospital surveys often have limited information about their validity and reliability as there is no standardized or commonly used instrument or protocol for sampling and administration [40]. Beattie *et al*.’s systematic review of general patient experience measures is a useful addition to research [22].

### Implications of the review

Concerns are raised by the fact that multiple PREMs have been developed for the same patient population with little concern given to the validation of the measures. It is unknown why researchers continue to develop poorly validated PREMs for the same population. Future research should consider drawing on the most promising existing PREMs as a starting point for the development of new measures. Existing instruments which have not been tested on certain criteria are not necessarily flawed, just untested. Such instruments may give useful information, but should be used with caution. Improving validation will allow them to provide more credible findings for use in future service improvement.

## Conclusion

Current PREMs for use within the ED were found to be adequately developed and offer promise for use within clinical settings. The review identified limited PREMs for emergency care service provision, with a low quality rating in terms of instrument performance. Without further work on validation, it is difficult to make recommendations for their routine use, as well as being difficult to draw credible findings from the results they produce. Further development and testing will make them more robust, allowing them to be better used within the population. Looking ahead, it would be of benefit to have a standardized sampling and administration protocol to allow easier development of PREMs specific to various areas and disease populations.

## Appendix 1 Search strategy

**Table:** Medline search strategy—search conducted 11/05/2015

#	Advanced search
1	‘patient experience*’.mp.
2	‘patient reported experience*’.mp.
3	Emergency Medical Services/
4	Psychometrics/
5	1 and 3 and 4
6	2 and 3 and 4
7	1 and 3
8	2 and 3
9	3 and 4
10	‘Measure*’ or ‘tool*’ or ‘instrument*’ or ‘survey*’ or ‘score*’ or ‘scale*’ or ‘questionnaire*’.mp.
11	1 and 3 and 8
12	2 and 3 and 8
13	‘emergency care’ or ‘unscheduled care’ or ‘unplanned care’.mp.
14	4 and 13

**Table:** Search results—May 2015

Database	Results
MEDLINE	52
CINHAL	63
PsycINFO	111
Scopus	157
Total Database results	383
Google Scholar	1
Picker website/emails	0
Secondary references	212
Total Identified Through Other Sources	213
Total	596
